# Sex differences in health-related quality of life and poverty risk among older people living with HIV in Spain: A cross-sectional study

**DOI:** 10.1371/journal.pone.0301335

**Published:** 2024-05-07

**Authors:** Néstor Nuño, Alberto Martínez, Susana Martínez, Marta Cobos, Juan Sebastián Hernández, Rosa Polo

**Affiliations:** 1 Division for Control of HIV, STIs, Viral Hepatitis and Tuberculosis, Spanish Ministry of Health, Madrid, Spain; 2 Working Group on HIV Treatments (gTt-HIV), Barcelona, Spain; North South University, BANGLADESH

## Abstract

**Background:**

Current antiretroviral therapies have increased the life expectancy of people living with HIV (PLHIV). There is, however, limited evidence regarding the health-related quality of life (HRQoL) and living conditions of older people living with HIV (OPLHIV) in Spain.

**Methods:**

We implemented a self-administered online questionnaire to identify sex differences in HRQoL and poverty risk among Spanish OPLHIV (PLHIV ≥50 years). Participants were contacted through non-governmental organisations. We used the standardised WHOQoL-HIV BREF questionnaire and the Europe 2020 guidelines to estimate HRQoL and poverty risk respectively. The statistical analysis included multivariable generalised linear models with potential confounding variables and robust estimates.

**Results:**

The study included 247 OPLHIV (192 men and 55 women). On the WHOQoL-HIV BREF questionnaire, men scored higher on 84% of items and in all six domains. Women had significantly lower HRQoL in five domains: physical health (β: -1.5; 95% CI: -2.5, -0.5; *p*: 0.002), psychological health (β: -1.0; 95% CI: -1.9, -0.1; *p*: 0.036), level of independence (β: -1.1; 95% CI: -1.9, -0.2; *p*: 0.019), environmental health (β: -1.1; 95% CI: -1.8, -0.3; *p*: 0.008), and spirituality/personal beliefs (β: -1.4; 95% CI: -2.5, -0.3; *p*: 0.012). No statistical differences were found in the domain of social relations. Poverty risk was considerable for both men (30%) and women (53%), but women were significantly more likely to experience it (OR: 2.9; 95% CI: 1.3, 6.5; *p*: 0.009).

**Conclusion:**

The aging of PLHIV is a public health concern. Our findings indicate that HRQoL and poverty risk among Spanish OPLHIV differ significantly by sex. Spain should, therefore, implement specific policies and interventions to address OPLHIV needs. The strategies must place a high priority on the reduction of sex inequalities in HRQoL and the enhancement of the structural conditions in which OPLHIV live.

## Introduction

Antiretroviral therapy (ART), has transformed the infection of the human immunodeficiency virus (HIV) into a chronic disease, increasing the life expectancy of people living with HIV (PLHIV) at comparable levels to those of the general population [1, 2]. Several studies found relatively positive health-related quality of life (HRQoL) for PLHIV in the ART era [3]. However, levels remained lower than those in the general population [4–6] and people with other chronic diseases [7].

Long-term HIV infection is a critical factor influencing HRQoL of PLHIV, as comorbidities, age-related impediments and non-infectious diseases are greatly prevalent among PLHIV [8, 9]. Nevertheless, multidisciplinary approaches emphasise the importance of addressing HIV-related impacts also from social and structural perspectives [10, 11]. According to various studies, HRQoL of PLHIV is negatively impacted by socio-demographic and psychological factors such as stigma, unwanted loneliness, depression, and financial stress [12–16].

Because of the early aging of the immune system caused by HIV infection, the literature consensus refers PLHIV who are ≥50 years as older people living with HIV (OPLHIV) [17]. As of 2016, there were 5.7 million OPLHIV in the world, and it was estimated a substantial increase in the coming decades [12, 14]. Several studies have confirmed that HRQoL of OPLHIV is lower than younger PLHIV [18–20]. Approximately 53% of PLHIV in Spain are OPLHIV, 15% are classified as frail, and 57% rated their health as poor or very poor in the previous year [21]. It should be noted, however, that none of these estimates take sex differences into account.

PLHIV experiences are closely associated with poverty, which encompasses material (e.g., the access to consumer goods), labour and economic dimensions. PLHIV at working age typically have greater difficulty finding employment due to HIV-related stigma. In turn, their jobs generally have worse conditions and salaries [22–24]. The structural inequalities that PLHIV experience over time result in deprivation, low social support and poor pensions in old age [25–28].

Several studies have described the HRQoL and structural living conditions of OPLHIV in different countries [29, 30]. However, these dimensions remain understudied in Spain, as only one study has assessed the HRQoL of OPLHIV, but without accounting for sex differences [18], and none has examined the structural conditions in which OPLHIV live. This cross-sectional study aims to identify sex differences in HRQoL and poverty risk among Spanish OPLHIV.

## Materials and methods

### Study design

We conducted a cross-sectional study between February and October 2022. A self-administered questionnaire that collected socio-demographic, health, lifestyle, and HRQoL information was implemented online. PLHIV ≥50 years in Spain who were able to make decisions autonomously were invited to participate in the study. Our study complies with the guidelines for strengthening the reporting of observational studies in epidemiology (STROBE). The STROBE is a 22-item checklist developed to facilitate the understanding of the methods, data analysis and results of observational studies [31].

### Sample size and participant selection

We calculated a non-probabilistic minimum sample size of 222 participants. Differences in HIV incidence in Spain were considered to estimate participants by sex. According to the last Spanish epidemiological surveillance report on HIV and AIDS, 84% of HIV cases were reported in men and 16% in women. For decades, HIV cases by sex have remained consistent. Applying a proportionate stratification criterion, the number of participants by sex was 186 men and 36 women. Sample size calculations are described in S1 File. Participants were selected by convenience in collaboration with gTt-VIH, one of the largest non-governmental organisations (NGOs) in Spain working with PLHIV. In order to identify participants who met the inclusion criteria, gTt-VIH collaborated with 19 smaller NGOs throughout the country.

### Outcomes and instruments

The main outcomes were self-reported HRQoL and poverty risk. We assessed HRQoL using the standardised WHOQoL-HIV BREF questionnaire. This instrument was specifically designed for PLHIV [32] and was recently validated in Spain [18]. We selected the WHOQoL-HIV BREF over other HRQoL instruments because of its briefness and robust psychometric properties [33]. In addition, the WHOQoL-HIV BREF has previously been applied to OPLHIV in Spain, France and Portugal [18, 34, 35]. The WHOQoL-HIV BREF questionnaire consists of 31 items grouped into six domains–physical health, psychological health, level of independence, social relations, environmental health, and spirituality/personal beliefs. The questions are scored on a five-point Likert scale between 1 and 5. For each domain, scores range from 4 to 20. Higher scores are associated with better HRQoL. Measurements were performed according to the questionnaire instructions. The Spanish validation of the WHOQoL-HIV BREF questionnaire showed an acceptable internal consistency with a Cronbach’s alpha coefficient of 0.70 and McDonald’s omega coefficient of 0.80 across most domains [18]. In our study, internal consistency was also acceptable, with a global Cronbach’s alpha coefficient of 0.86 and a global McDonald’s omega coefficient of 0.88. The psychometric properties of the WHOQoL-HIV BREF questionnaire by domain are presented in S2 File.

Poverty risk was defined, according to the Europe 2020 strategy guidelines [36], as a person with low labour intensity, low income or severe material deficiencies. People with low work intensity are those with an employment intensity below 20% of their total work potential in the year prior to the questionnaire. Work potential is the quotient between the number of months in which the person worked, and the number of months the person did not work without force majeure impediments. This indicator is not applicable to people >60 years. People with low income are those who received incomes below 60% of the national median equivalent income in the previous year. According to the Spanish National Institute of Statistics, the equivalent median income in 2020 was €16,339 for men and €15,804 for women [37]. People with severe material deprivation lack at least 4 (out of 9) dimensions described in the Europe 2020 strategy [38].

### Data collection and analysis

Data was collected using the open-source app Kobo Toolbox (KoBo, Inc.). To reduce missing data, all questions were mandatory, but participants were permitted to decline to answer or select a neutral response. In order to prevent the same user from submitting the questionnaire twice, we programmed the tool to accept only one response per electronic device. Upon completing data collection, we reviewed and cleaned the data for errors and inconsistencies in the responses. We used descriptive statistics (e.g., frequency tables for categorical variables, and mean and standard deviation for numerical variables) to present socio-demographic, health, and lifestyle information. To identify differences across numerical variables by sex, we used the Student’s t-test or its non-parametric alternative, the Mann-Whitney test. In the case of categorical variables, we applied the chi-square test or Fischer’s exact test.

To assess sex differences in HRQoL domains and poverty risk, we used univariable generalised linear models with sex (0 = men; 1 = women) as a predictor variable. Additionally, we designed multivariable generalised linear models incorporating potential confounding variables derived from the literature. Multivariable models for HRQoL domains included the following predictor variables: sex, age, years since HIV diagnosis, being on ART (0 = no; 1 = yes), number of comorbidities experienced in the previous year or chronic, and presence of self-reported mobility impairments (0 = no; 1 = yes) and mental disorders (0 = no; 1 = yes) experienced in the previous year or chronic. The full list of comorbidities included: Cardiovascular disease, stroke, diabetes mellitus, respiratory diseases (e.g., pulmonary embolism, coronary angioplasty), hypertension, cancer/tumor, sexually transmitted infection, and dementia/Alzheimer. The multivariable model for poverty risk included the following predictor variables: sex, region of origin (0 = other; 1 = Spain), years since HIV diagnosis, employment status (0 = other; 1 = employed), education level (0 = mandatory; 1 = higher), relationship status (0 = single; 1 = stable partner), number of comorbidities experienced in the previous year or chronic, and presence of self-reported mobility impairments and mental disorders experienced in the previous year or chronic. We used robust estimates of standard errors to calculate confidence intervals. The statistical analysis was performed using R 3.6 (R Project for statistical computing).

### Ethics

Participation in the study was voluntary and no financial compensation was offered to encourage participation. The researchers did not receive any compensation for their work on the study. Due to the observational and non-invasive nature of the study, participation did not entail any risk.

Neither the questionnaires nor informed consent forms collected any personal information as some PLHIV in Spain refuse to disclose their serological status for HIV publicly due to HIV-related stigma [39]. The ethical board of La Princesa Hospital (Madrid) exempted the study from review. The study was conducted in accordance with the Helsinki Declaration. All participants signed an informed consent form prior to completing the questionnaire.

## Results

### Participant characteristics

The questionnaire was completed by 257 participants, but 10 participants were excluded from the final analysis: n = 3 did not indicate their age and n = 7 were ≤50 years. The final analysis included 247 participants: 192 men (78%) and 55 women (22%). Seventy-three percent of male (n = 140) and 85% of female (n = 47) participants were ≤60 years.

**Fig 1:**
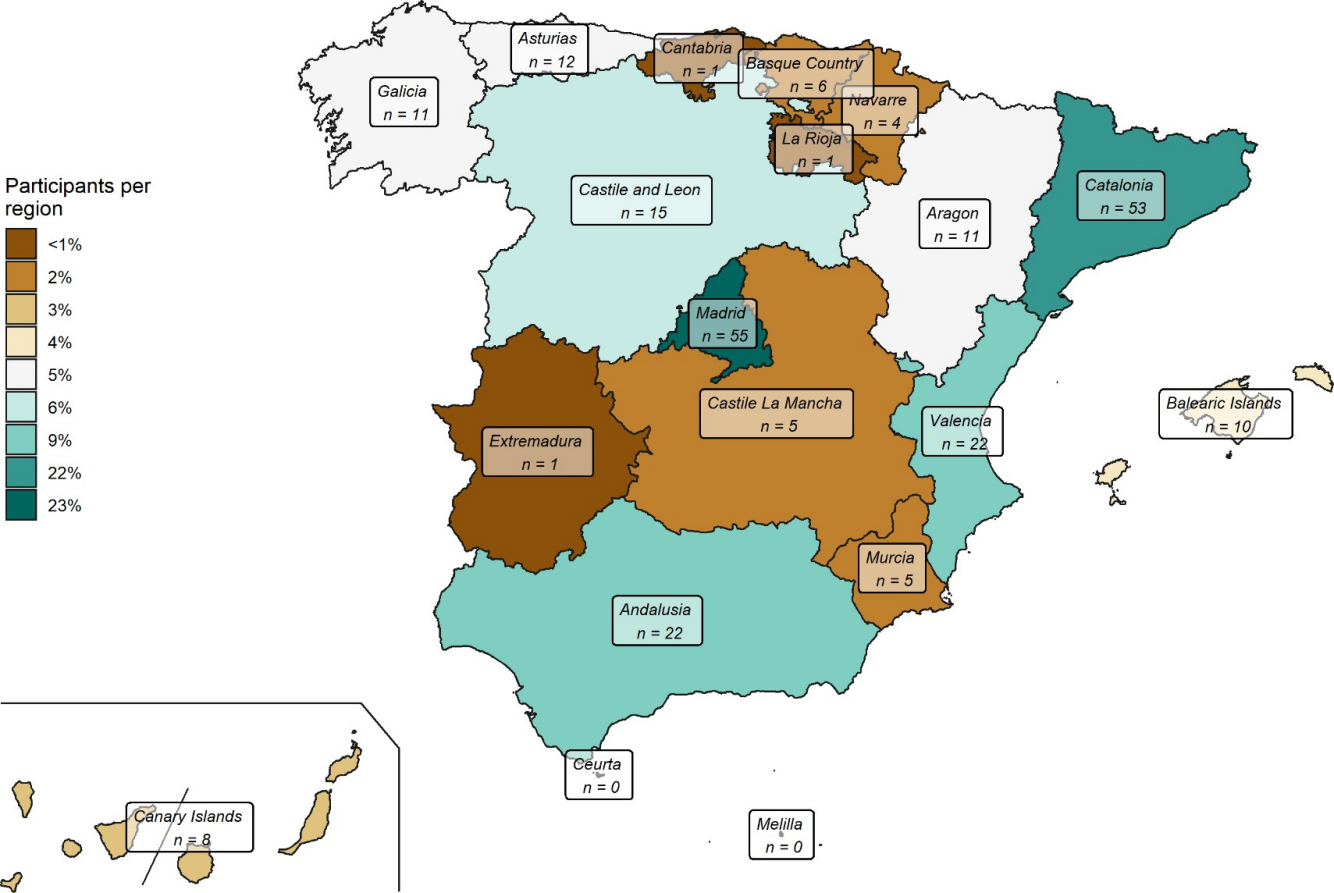
Distribution of study participants by region. No participants from the autonomous cities of Ceuta and Melilla were registered. The image was created using freely accesible data from the Spanish National Geographic Institute and is licensed under the Creative Commons Attribution License (CC BY 4.0).

In the study, 89% of Spanish regions were represented, with the majority of participants hailing from Madrid (23%), Catalonia (22%), Valencia (9%), and Andalusia (9%) regions. Five participants (2%) did not indicate their residence region. Fig 1 represents the distribution of participants by region.

Table 1 summarises the sociodemographic and health characteristics of participants. The mean age was 56.9 (SD = 5.1) years, the majority (89%) was born in Spain, and 83% held a higher education degree, with a mean of 14.5 (SD = 3.9) years of study. Fifty-seven percent of participants identified themselves as homosexuals, 41% had a stable partner and 56% were employed. Concerning health characteristics, 56% of participants self-reported being in good health in the previous year. The average number of years since HIV diagnosis was 21.2 (SD = 10.5). Almost all participants had an undetectable viral load (97%) and were on ART (99%). Seventy-four percent of HIV infections result from unprotected sex. Mobility impairments (21%), mental disorders (20%) and renal failure (5%) were the most common self-reported comorbidities experienced in the previous year or chronic. An average of 1.2 (SD = 1.1) comorbidities were reported by participants, 35% smoked, 79% consumed alcohol, 82% exercised regularly, and 41% had used some drugs during the previous month. The full lust of drugs included: erection enhancing drugs, cannabis, hashish, cocaine, MDMA, amphetamines, ketamine, GBH, methamphetamine, mephedrone, and poppers.

**Table 1:** Socio-demographic, health and lifestyle characteristics of study participants by sex.

	Total		Men		Women		*p-*value
	N	% (n) / Mean (SD)	N	% (n) / Mean (SD)	N	% (n) / Mean (SD)	
*Socio-demographic characteristics*	247		192		55		
Age (years)		56.9 (5.1)		57.1 (5.4)		56.3 (4.0)	0.606
Birthplace (Spain)		88.7% (219)		86.5% (166)		96.4% (53)	0.041
Degree in education (higher education)^a^		82.6% (204)		88.3% (165)		64.5% (36)	<0.001
Education (number of years of study)^b^		14.5 (3.9)		15.1 (3.6)		12.4 (4.2)	<0.001
Sexual orientation (homosexual)	244	56.6% (138)	190	70.5% (134)	54	7.4% (4)	<0.001
Civil state (stable partner)	246	41.1% (101)	191	43.5% (83)		32.7% (18)	0.154
Working situation (salaried employee)	245	56.3% (138)	190	59.0% (112)		47.3% (26)	0.124
*Health characteristics*	247		192		55		
Self-reported good health (previous year)		55.6% (138)		62.0% (119)		34.6% (19)	<0.001
Years since HIV diagnosis	236	21.2 (10.5)	181	19.2 (10.0)		28.0 (9.4)	<0.001
Viral load (undetectable)	243	97.1% (236)	189	97.9% (185)	54	94.4% (51)	0.183
ART	246	99.2% (244)	191	99.5% (190)		98.2% (54)	0.346
HIV infection route (unprotected sex)	245	73.5% (180)	190	80.0% (152)		50.9% (28)	<0.001
Mobility impairments^c^	245	21.2% (52)	191	17.3% (33)	54	35.2% (19)	0.004
Mental disorders^c^	245	19.6% (48)	191	19.4% (37)	54	20.4% (11)	0.870
Renal failure^c^	245	4.5% (11)	191	2.1% (4)	54	13.0% (7)	0.001
Comorbidities (total number)^c^	245	1.2 (1.1)	191	1.2 (1.1)	54	1.4 (1.2)	0.176
*Lifestyle characteristics*	247		192		55		
Smoke	246	35.4% (87)	191	34.0% (65)		40.0% (22)	0.415
Alcohol consumption	245	79.2% (194)	190	81.6% (155)		70.9% (39)	0.086
Regular physical activity		82.2% (203)		83.9% (161)		76.4% (42)	0.201
Drugs consumption (previous month)		41.3% (102)		44.8% (86)		29.1% (16)	0.037

SD: standard deviation. ART: antiretroviral treatment. *p*-values calculated applying the chi-square, Fisher´s exact and Mann-Whitney tests.

^a^Higher education in Spain is not compulsory and may require a fee.

^b^It includes the following categories: primary education (6 years); secondary education (10 years), baccalaureate (12 years); higher education, non-university (14 years); higher education, university (17 years); and higher education, master/doctorate (20 years).

^c^Self-reported in the previous year or chronic.

As for socio-demographic characteristics, women had significantly lower levels of education (*p*<0.001), fewer years of study (*p*<0.001), and identified themselves as homosexuals less often (*p*<0.001). In contrast, women were born primarily in Spain (*p* = 0.041). Across health and lifestyle characteristics, women had significantly lower perceptions of good health (*p*<0.001), higher years since HIV diagnosis (*p*<0.001), lower percentages of infection through unprotected sex (*p*<0.001), greater self-reported mobility impairments (*p* = 0.004) and renal failure (*p* = 0.001), and less drugs consumed (*p* = 0.037) (Table 1).

### Health-related quality of life

In the WHOQoL-HIV BREF questionnaire, men scored higher than women on 26 of the 31 items (84% of the total). In two items (overall general health, and information for daily living) men and women scored similarly. Women outperformed men on three items: social support, personal relationships, and death and dying. Men obtained higher scores in the six domains of the WHOQoL-HIV BREF questionnaire (difference range: 2–14%) (Table 2).

**Table 2:** Results of the WHOQoL-HIV BREF questionnaire by sex.

		Total (N = 247)	Men (N = 192)		Women (N = 55)	
		Mean (SD)	Mean (SD)		Mean (SD)	
*Overall HRQoL/General Health* ^a^		12.6 (2.7)	12.8 (2.6)		11.9 (3.1)	
Overall HRQoL		3.4 (0.9)	3.5 (0.9)		3.0 (0.9)	
Overall general health		2.9 (1.0)	2.9 (1.0)		2.9 (1.0)	
*Physical health*	13.0 (3.4)			13.4 (3.3)	11.5 (3.4)
Pain and discomfort^b^	3.5 (1.2)			3.6 (1.2)	3.1 (1.2)
Symptoms of HIV^b^	3.8 (1.2)			3.9 (1.2)	3.3 (1.2)
Energy and fatigue	3.1 (1.0)			3.2 (0.9)	2.9 (1.0)
Sleep and rest	2.6 (1.1)			2.7 (1.1)	2.2 (1.0)
*Psychological health*	12.5 (3.1)			12.7 (3.1)	11.8 (2.8)
Positive feelings	3.3 (1.0)			3.3 (1.0)	3.2 (1.0)
Concentration ability	3.0 (1.0)			3.0 (0.9)	2.8 (1.1)
Body image self-acceptance	3.3 (1.0)			3.4 (1.0)	3.0 (1.1)
Self-satisfaction	3.2 (1.0)			3.3 (1.0)	3.1 (1.0)
Negative feelings^b^	2.9 (1.0)			2.9 (1.0)	2.7 (0.9)
*Level of independence*	13.2 (3.2)			13.5 (3.1)	12.2 (3.2)
Dependence on medication^b^	3.0 (1.3)			3.1 (1.3)	2.8 (1.2)
Mobility	3.2 (1.0)			3.3 (1.0)	2.9 (1.0)
Activities of daily living	3.1 (1.0)			3.2 (1.0)	2.8 (1.1)
Work capacity	3.9 (1.0)			3.9 (0.9)	3.8 (1.1)
*Social relations*	12.5 (3.2)			12.5 (3.3)	12.3 (2.9)
Social support	3.3 (1.1)			3.2 (1.1)	3.6 (1.0)
Sexual satisfaction	2.4 (1.1)			2.5 (1.1)	2.0 (1.1)
Personal relationships	3.1 (1.0)			3.1 (1.0)	3.2 (1.0)
Social inclusion	3.7 (0.9)			3.7 (0.9)	3.5 (0.9)
*Environmental health*	13.9 (2.6)			14.2 (2.6)	13.2 (2.5)
Physical safety and security	3.1 (0.9)			3.2 (0.9)	3.0 (0.8)
Physical environment	3.5 (0.8)			3.5 (0.8)	3.4 (0.9)
Financial resources	3.3 (1.0)			3.4 (1.0)	2.8 (0.9)
Information for daily living	3.7 (0.9)			3.7 (0.9)	3.7 (0.8)
Participation in leisure activities	3.4 (1.0)			3.5 (1.0)	3.2 (1.0)
Home environment	3.7 (1.0)			3.7 (1.0)	3.6 (1.1)
Accessibility of health services	3.5 (1.0)			3.6 (1.0)	3.3 (1.1)
Transport	3.6 (1.0)			3.6 (0.9)	3.3 (1.1)
*Spirituality/personal beliefs*	12.9 (3.7)			13.2 (3.8)	12.1 (3.1)
Personal life meaning	3.4 (1.1)			3.4 (1.2)	3.2 (1.0)
Forgiveness and blame^b^	3.1 (1.6)			3.3 (1.6)	2.6 (1.5)
Concerns about the future^b^	3.0 (1.2)			3.0 (1.2)	2.8 (1.1)
Death and dying^b^	3.5 (1.2)			3.4 (1.2)	3.5 (1.1)

SD: standard deviation.

aAccording to the instructions for the questionnaire, these items do not belong to any domain.

bReversed items recorded.

### Poverty risk

A major material deprivation among participants was the inability to pay for unexpected expenses (37%), the capacity to afford a car (29%), and the inability to take an annual week off (23%). The average number of months participants were unemployed was 3.9 (SD = 5.0), compared to 7.6 (SD = 5.0) months they worked. Approximately 33% of respondents experienced difficulties making ends meet, with the median income reported at €20,000 (IQR = €14,000, €34,000). In total, 13% of participants suffered severe material deprivation, 38% had low work intensity, and 21% earned a low income. The percentage of participants at risk of poverty was 35% (Table 3).

**Table 3:** Material, labour and economic deprivation and poverty risk by sex.

	Total		Men		Women		*p-*value
	N	% (n) / Mean (SD) / Median (IQR)	N	% (n) / Mean (SD) / Median (IQR)	N	% (n) / Mean (SD) / Median (IQR)	
*Material deprivation* ^a^	247		192		55		
Consume meat or fish (each two days)	244	5.7% (14)	189	5.8% (11)		5.5% (3)	0.918
Car	235	28.9% (68)	181	24.3% (44)	54	44.4% (24)	0.004
Maintain an adequate household temperature	243	15.2% (37)	188	11.2% (21)		29.1% (16)	0.001
Pay for unexpected expenses^b^	239	36.8% (88)	186	30.1% (56)	53	60.4% (32)	<0.001
Washing machine	245	2.0% (5)	190	2.1% (4)		1.8% (1)	1.000
At least one payment delay (previous year)	242	14.1% (34)	188	13.3% (25)	54	16.7% (9)	0.530
Telephone	246	0.8% (2)	191	1.0% (2)		0.0% (0)	1.000
Television	246	4.1% (10)	191	3.7% (7)		5.5% (3)	0.697
Have a week of holidays (per year)	174	23.0% (40)	138	18.8% (26)	36	38.9% (14)	0.011
*Labour deprivation* ^c^	187		140		47		
Unemployment (months previous year)^d^	167	3.9 (5.0)	127	3.2 (4.7)	40	6.0 (5.3)	0.007
Employment (months previous year)	167	7.6 (5.0)	127	8.2 (4.8)	40	5.7 (5.3)	0.019
*Economic deprivation*	247		192		55		
Total income (previous year)^e^	161	20 (14, 34)	130	23 (14, 37)	31	17 (8, 20)	0.001
Make ends meet (with any difficulty)		33.2% (82)		27.6% (53)		52.7% (29)	<0.001
*Poverty risk estimation*	247		192		55		
Severe material deprivation		12.6% (31)		10.9% (21)		18.2% (10)	0.153
Low work intensity^b^	167	38.3% (64)	127	31.5% (40)	40	60.0% (24)	0.001
Low income	187	20.9% (39)	151	17.2% (26)	36	36.1% (13)	0.012
Poverty risk		35.2% (87)		30.2% (58)		52.7% (29)	0.002

SD: standard deviation; IQR: interquartile range. *p*-values calculated applying the chi-square, Fisher´s exact and Mann-Whitney tests.

^a^Difficulties to achieve/afford/have.

^b^Of at least €750.

^c^Participants >60 years are excluded.

^d^Without force majeure impediments.

^e^In thousands of euros.

In general, women reported more material, labour, and economic deprivation than men. We found statistically differences in the capacity to afford a car (*p* = 0.004), keeping an adequate household temperature (*p* = 0.001), paying unexpected expenses (*p*<0.001), and having an annual week of holidays (*p* = 0.011). Similarly, women reported fewer months working (*p* = 0.019) and less income (*p* = 0.001), as well as a higher percentage of unemployed months (*p* = 0.007) and difficulty making ends meet (*p*<0.001). The prevalence of low working intensity (*p* = 0.001), low income (*p* = 0.012) and poverty risk (*p* = 0.002) was also higher among women (Table 3).

### Sex differences in health-related quality of life and poverty risk

The multivariable statistical analysis showed that women had significantly lower HRQoL in five domains: physical health (β: -1.5; 95% CI: -2.5, -0.5; *p*: 0.002), psychological health (β: -1.0; 95% CI: -1.9, -0.1; *p*: 0.036), level of independence (β: -1.1; 95% CI: -1.9, -0.2; *p*: 0.019), environmental health (β: -1.1; 95% CI: -1.8, -0.3; *p*: 0.008), and spirituality/personal beliefs (β: -1.4; 95% CI: -2.5, -0.3; *p*: 0.012). No statistical differences were found in the domain of social relations. Women were 2.9 times more likely to experience poverty risk than men (OR: 2.9; 95% CI: 1.3, 6.5; *p*: 0.009) (Table 4).

**Table 4:** Statistical models for assessing sex differences in HRQoL and poverty risk.

	Unadjusted model^a^			Adjusted model^b^		
	Mean difference	95% CI	*p*-value	Mean difference	95% CI	*p*-value
Physical health	-1.9	-2.9, -0.9	<0.001	-1.5	-2.5, -0.5	0.002
Psychological health	-0.9	-1.8, -3e^-2^	0.027	-1.0	-1.9, -0.1	0.036
Level of independence	-1.3	-2.2, -0.3	0.008	-1.1	-1.9, -0.2	0.019
Social relations	-0.2	-1.1, 0.6	0.585	-0.4	-1.3, 0.5	0.378
Environmental health	-1.0	-1.8, -0.2	0.010	-1.1	-1.8, -0.3	0.008
Spirituality/personal beliefs	-1.1	-2.1, -0.1	0.024	-1.4	-2.5, -0.3	0.012
	Unadjusted model^a^			Adjusted model^c^		
	OR	95% CI	*p*-value	OR	95% CI	*p*-value
Poverty risk	2.6	1.4, 4.8	0.003	2.9	1.3, 6.5	0.009

CI: confidence interval; OR: odd ratio.

aEstimated by generalised linear models with robust estimates.

bEstimated by generalised linear models with robust estimates adjusted by age, years since HIV diagnosis, being on ART, number of comorbidities and presence of self-reported mobility impairments and mental health disorders.

cEstimated by generalised linear models with robust estimates adjusted by region of origin, years since HIV diagnosis, employment status, education level, relationship status, number of comorbidities and presence of self-reported mobility impairments and mental health disorders.

## Discussion

We conducted a cross-sectional study with 247 Spanish older people living with HIV (OPLHIV) to identify sex differences in their health-related quality of life (HRQoL) and poverty risk. HRQoL was determined using the standardised WHOQoL-HIV BREF questionnaire, which was recently validated in Spain [18]. Poverty risk was estimated based on the guidelines of the Europe 2020 strategy, which is the current methodology used in the European Union. This is, to our knowledge, one of the few studies that assess HRQoL and poverty risk for OPLHIV in Spain using standardised instruments and accounting for sex differences.

Our results showed that male and female participants differed in terms of socio-demographic, health, and structural characteristics. In general, women defined themselves as homosexuals less often, and had lower levels of education (degrees completed and years of study), income, work intensity, drug consumption, and self-reported perception of good health in the previous year. Conversely, women had greater self-reported mobility impairments and renal failure, more years since HIV diagnosis, higher levels of material deprivation, and more difficulties to make ends meet. These characteristics are consistent with those of previous studies with OPLHIV [40–42].

We found substantial differences in HRQoL by sex. Women had significantly lower HRQoL in the domains of physical health, psychological health, level of independence, environmental health, and spirituality/personal beliefs. Recent studies have reported poor physical health among women living with HIV (WLHIV). In England, Brañas et al., found that WLHIV ≥50 years had a lower HRQoL than their male counterpart [42]. However, the study was limited to 100 participants (27 women). Additionally, in a cohort of 1,000 OPLHIV (25% women) in Italy it was found that WLHIV had lower physical strength, increased frailty and lower HRQoL than men living with HIV (MLHIV) [43]. Sex differences in psychological health are common among WLHIV, as they generally suffer from depression, anxiety and post-traumatic stress [44]. A study with 357 WLHIV in Ethiopia found that anxiety and depression were present in around 30% of the participants. Factors such as low education, divorce, unemployment or financial burdens were negatively associated with depression. However, the study did not focus on OPLHIV exclusively [45]. In a recent review, Waldron et al., showed that WLHIV generally have strong histories of physical and sexual abuse, caregiving stress, elevated internalised stigma, and a wide range of barriers to care. As a result, there is a high prevalence of depression, anxiety, and trauma-related mental health issues among WLHIV. For example, WLHIV could be up to four times more likely to be diagnosed with major depressive disorder and experience more severe depressive symptom than HIV-seronegative women [46]. However, the review did not focus on sex differences, nor specifically on OPLHIV. The observed living and structural conditions of women in our study may be related to sex differences in the level of independence and environmental health domains, as these two domains include items relating to work capacity, physical environment, living conditions, and economic resources. Other studies also indicate that WLHIV generally live in poorer conditions and have lower socioeconomic status than MLHIV. The study conducted by Kalichman et al., found that income inequality, internalised stigma, and enacted stigma were significantly associated with HIV suppression among WLHIV (but not in MLHIV). However, the study did not focus on OPLHIV exclusively [47]. Likewise, a qualitative study among WLHIV (89% African American) found that that poverty, unemployment, limited access to healthcare resources and stigma impacted negatively on their health and ability to engage in HIV care [48]. This study, however, did not focus on sex differences, nor specifically on OPLHIV.

Our results are also consistent with recent studies in Spain. In a study conducted by Fumaz et al., WLHIV had significantly lower physical function and psychological health than MLHIV, although the sample did not include OPLHIV exclusively [49]. Fuster-Ruiz de Apodaca et al. found that WLHIV scored lower in 61% of items and the six domains of the WHOQoL-HIV BREF questionnaire. Although 38% of participants (n = 549) were OPLHIV, sex differences in HRQoL were only examined for the total sample [18]. Ruiz-Algueró et al. found that 63% of OPLHIV described their health as good in the previous year, although the results were not disaggregated by sex [50]. In our study, we found a similar result with 57% of participants defining their health as good in the previous year. Finally, other studies conducted with OPLHIV in Germany, Brazil, the United States, Italy and Portugal concluded that WLHIV tend to have a worse HRQoL than MLHIV [19, 41–43, 51].

In our study, we found that 53% of women were at risk of poverty and that they were more likely to suffer it than men. Despite this difference, it is important to remark that men were also at high poverty risk (30%). Other studies have shown that OPLHIV are particularly vulnerable to poverty and inequality, although none of them specifically address sex differences. In Canada, Sok et al. reported that 87% of 496 PLHIV did not have access to basic needs (e.g., clothing, food). Particularly among OPLHIV, unmet basic needs were associated with poorer physical and psychological health [30]. Similarly, Hessol et al. found that 32% of 230 OPLHIV in the United States lacked regular access to healthy food. In addition, food insecurity was associated with alcohol consumption, sedentary lifestyles, and depression [29]. Finally, a study conducted in the United Kingdom with 307 OPLHIV found that 58% were living at or below the poverty line, only 45% (of those aged 50–64 years) were economically active, and 32% were dependent on social benefits [52].

We identified some limitations in our study. Although our estimates of poverty risk were consistent with previous studies, the impact of the SARS-CoV-2 pandemic on labour and economics could have caused an overestimation of the outcome. For example, the pandemic caused a substantial negative impact on the Spanish economy, with unemployment rates increasing to 36% in April and May 2020 [53]. In contrast, we also observed response patterns that were associated with a potential underestimation of our poverty risk estimates, as questions related to economic, employment, and material deficiencies had a higher proportion of missing responses. There is evidence that socioeconomically disadvantaged groups are more likely to leave these questions unanswered [54, 55] Nevertheless, we do not believe that this missingness alters the interpretation of our results since the proportion of missing responses was similar across sexes. Last but not least, we are unable to generalise our results to the population of OPLHIV in Spain due to the sample size and the selection of participants. Our findings, however, are consistent with those of other previous studies conducted with OPLHIV in Spain.

Overall, our results can be helpful in identifying OPLHIV needs in Spain. According to a recent series published in The Lancet Healthy Longevity, new research areas on HIV and aging are needed to address the complex challenges that OPLHIV face [56]. Commonly, older adults have been marginalised and underrepresented in research [57, 58]. Now it is time to resolve this trend and address how modern societies can meet the needs of elderly populations. There are several factors related to HIV and aging that must be taken into account. In this regard, it will be difficult to achieve tangible and transformative improvements for OPLHIV without gender-sensitive policies and interventions.

## Conclusion

We conducted a cross-sectional study among 247 Spanish older persons living with HIV (192 women and 55 men) in order to identify sex differences in health-related quality of life and poverty risk. In general, women had a significantly lower health-related quality of life and a higher poverty risk than men. In light of these results, future gender-sensitive policies are required to improve living conditions and provide comprehensive care for older people living with HIV in Spain.

## Supporting information

S1 File Sample size calculation. (PDF)

S2 File Psychometric properties of the WHOQoL-HIV BREF questionnaire. (PDF)

## References

[1] Nakagawa F, Lodwick RK, Smith CJ, Smith R, Cambiano V, Lundgren JD, et al. (2012). Projected life expectancy of people with HIV according to timing of diagnosis. AIDS. 2012; 26(3): 335–343. doi: 10.1097/QAD.0b013e32834dcec9 22089374

[2] The Antiretroviral Therapy Cohort Collaboration. Survival of HIV-positive patients starting antiretroviral therapy between 1996 and 2013: a collaborative analysis of cohort studies. The Lancet HIV. 2017; 4(8): e349–e356. doi: 10.1016/S2352-3018(17)30066-8 28501495 PMC5555438

[3] Aragonés-López C, Pérez-Ávila J, Smith Fawzi MC, Castro A. Quality of life of people with HIV/AIDS receiving antiretroviral therapy in Cuba: a cross-sectional study of the national population. American Journal of Public Health. 2012; 102(5): 884–892. doi: 10.2105/AJPH.2011.300450 22420793 PMC3483902

[4] Lam JO, Hou CE, Hojilla JC, Anderson AN, Gilsanz P, Alexeeff SE, et al. Comparison of dementia risk after age 50 between individuals with and without HIV infection. AIDS. 2021; 35(5): 821–828. doi: 10.1097/QAD.0000000000002806 33394681 PMC7969394

[5] Miners A, Phillips A, Kreif N, Rodger A, Speakman A, Fisher M, et al. Health-related quality-of-life of people with HIV in the era of combination antiretroviral treatment: a cross-sectional comparison with the general population. The Lancet HIV. 2014; 1(1): e32–40. doi: 10.1016/S2352-3018(14)70018-9 26423814

[6] Pedersen KK, Eiersted MR, Gaardbo JC, Pedersen M, Gerstoft J, Troseid M, et al. Lower Self-Reported Quality of Life in HIV-Infected Patients on cART and With Low Comorbidity Compared With Healthy Controls. Journal of Acquired Immune Deficiency Syndromes. 2015; 70(1): 16–22. doi: 10.1097/QAI.0000000000000697 26017659

[7] Engelhard EAN, Smit C, van Dijk PR, Kuijper TM, Wermeling PR, Weel AE, et al. Health-related quality of life of people with HIV: an assessment of patient related factors and comparison with other chronic diseases. AIDS. 2018; 32(1): 103–112. doi: 10.1097/QAD.0000000000001672 29112062

[8] Guaraldi G, Orlando G, Zona S, Menozzi M, Carli F, Garlassi E, et al. Premature age-related comorbidities among HIV-infected persons compared with the general population. Clinical Infectious Diseases. 2011; 53(11): 1120–1126. doi: 10.1093/cid/cir627 21998278

[9] Morales DR, Moreno-Martos D, Matin N, McGettigan P. Health conditions in adults with HIV compared with the general population: A population-based cross-sectional analysis. EClinicalMedicine. 2022; 47: 101392. doi: 10.1016/j.eclinm.2022.101392 35497059 PMC9046106

[10] Farmer P. On Suffering and Structural Violence: A View from Below. Race/Ethnicity: Multidisciplinary Global Contexts. 2009; 3(1): 11–28.

[11] Singer M, Clair S. Syndemics and public health: reconceptualizing disease in bio-social context. Medical Anthropology Quarterly. 2003; 17(4): 423–441. doi: 10.1525/maq.2003.17.4.423 14716917

[12] Autenrieth CS, Beck EJ, Stelzle D, Mallouris C, Mahy M, Ghys P. Global and regional trends of people living with HIV aged 50 and over: Estimates and projections for 2000–2020. PLoS One. 2018; 13(11): e0207005. doi: 10.1371/journal.pone.0207005 30496302 PMC6264840

[13] Fuster-Ruiz de Apodaca MJ, Molero F, Holgado FP, Mayordomo S. Enacted and internalized stigma and quality of life among people with HIV: the role of group identity. Quality of Life Research. 2014; 23(7): 1967–1975. doi: 10.1007/s11136-014-0653-4 24585185

[14] Hsieh E, Polo R, Qian H, Fuster-Ruiz de Apodaca MJ, del Amo J. Intersectionality of stigmas and health-related quality of life in people ageing with HIV in China, Europe, and Latin America. The Lancet Healthy Longevity. 2022; 3(3): e206–e215. doi: 10.1016/S2666-7568(22)00003-4 36098292

[15] Nobre N, Pereira M, Roine RP, Sintonen H, Sutinen J. Factors associated with the quality of life of people living with HIV in Finland. AIDS Care. 2017; 29(8): 1074–1078. doi: 10.1080/09540121.2017.1281879 28110552

[16] Vincent W, Fang X, Calabrese SK, Heckman TG, Sikkema KJ, Hansen NB (2017). HIV-related shame and health-related quality of life among older, HIV-positive adults. Journal of Behavioral Medicine. 2017; 40(3): 434–444. doi: 10.1007/s10865-016-9812-0 27904976 PMC5407911

[17] Greene M, Justice AC, Lampiris HW, Valcour V. Management of human immunodeficiency virus infection in advanced age. JAMA. 2013; 309(13): 1397–1405. doi: 10.1001/jama.2013.2963 23549585 PMC3684249

[18] Fuster-Ruiz de Apodaca MJ, Laguía A, Safreed-Harmon K, Lazarus JV, Cenoz S, Del Amo J. Assessing quality of life in people with HIV in Spain: psychometric testing of the Spanish version of WHOQOL-HIV-BREF. Health Quality Life Outcomes. 2019; 17(1): 144. 10.1186/s12955-019-1208-8 PMC6700970 31426799

[19] Monteiro F, Canavarro MC, Pereira M. Factors associated with quality of life in middle-aged and older patients living with HIV. AIDS Care. 2016; 28 Suppl 1(sup1): 92–98. doi: 10.1080/09540121.2016.1146209 26881294 PMC4828599

[20] Skevington SM. Is quality of life poorer for older adults with HIV/AIDS? International evidence using the WHOQOL-HIV. AIDS Care. 2012; 24(10): 1219–1225. doi: 10.1080/09540121.2012.661838 22428745

[21] CNE, ISCII, PNS. Encuesta Hospitalaria de pacientes con infección por el VIH. Resultados 2021. Análisis de la evolución 2006–2021. 2021 [Cited 2023 January 20]. Available from: https://www.isciii.es/QueHacemos/Servicios/VigilanciaSaludPublicaRENAVE/EnfermedadesTransmisibles/Documents/VIH/informes%20encuesta%20hospitalaria%20anteriores/Informe%20Encuesta%20hospitalaria_2006_2021_def.pdf

[22] Annequin M, Lert F, Spire B, Dray-Spira R, Anrs-Vespa Study Group. Increase in Unemployment over the 2000’s: Comparison between People Living with HIV and the French General Population. PLoS One. 2000; 11(11): e0165634–e0165634. 10.1371/journal.pone.0165634 PMC5096670 27814374

[23] Conover C J, Arno P, Weaver M, Ang A, Ettner SL. Income and employment of people living with combined HIV/AIDS, chronic mental illness, and substance abuse disorders. Journal of Mental Health Policy and Economics. 2006; 9(2): 71–86. 17007485

[24] Peña Longobardo LM, Oliva-Moreno J. Differences in labour participation between people living with HIV and the general population: Results from Spain along the business cycle. PLoS One. 2018; 13(4): e0195735. doi: 10.1371/journal.pone.0195735 29684076 PMC5912724

[25] Blalock AC, McDaniel JS, Farber EW. Effect of employment on quality of life and psychological functioning in patients with HIV/AIDS. Psychosomatics. 2002; 43(5): 400–404. doi: 10.1176/appi.psy.43.5.400 12297609

[26] Liu Y, Canada K, Shi K, Corrigan P. HIV-related stigma acting as predictors of unemployment of people living with HIV/AIDS. AIDS Care. 2012; 24(1): 129–135. doi: 10.1080/09540121.2011.596512 21777074

[27] Rüütel K, Pisarev H, Loit H, Uusküla A. Factors influencing quality of life of people living with HIV in Estonia: a cross-sectional survey. Journal of the International AIDS Society. 2008; 12, 13–13. 10.1186/1758-2652-12-13 PMC2717916 19607721

[28] Zeng C, Guo Y, Hong YA, Gentz S, Zhang J, Zhang H, et al. Differential effects of unemployment on depression in people living with HIV/AIDS: a quantile regression approach. AIDS Care. 2019; 31(11): 1412–1419. doi: 10.1080/09540121.2019.1587366 30835499

[29] Hessol NA, Zepf R, Zobell E, Weiser SD, John MD. Food Insecurity and Aging Outcomes in Older Adults Living with HIV. AIDS and Behavior. 2017; 21(12): 3506–3514. doi: 10.1007/s10461-017-1838-y 28653132

[30] Sok P, Gardner S, Bekele T, Globerman J, Seeman MV, Greene S, et al. Unmet basic needs negatively affect health-related quality of life in people aging with HIV: results from the Positive Spaces, Healthy Places study. BMC Public Health. 2018; 18(1): 644. doi: 10.1186/s12889-018-5391-z 29783965 PMC5963101

[31] von Elm E, Altman DG, Egger M, Pocock SJ, Gøtzsche PC, Vandenbroucke JP, et al. The Strengthening the Reporting of Observational Studies in Epidemiology (STROBE) statement: guidelines for reporting observational studies. Journal of Clinical Epidemiology. 2008; 61(4): 344–9. doi: 10.1016/j.jclinepi.2007.11.008 18313558

[32] O’Connell KA, Skevington SM. An international quality of life instrument to assess wellbeing in adults who are HIV-positive: a short form of the WHOQOL-HIV (31 items). AIDS and Behavior. 2012; 16(2): 452–460. doi: 10.1007/s10461-010-9863-0 21181253

[33] Cooper V, Clatworthy J, Harding R, Whetham J. Measuring quality of life among people living with HIV: a systematic review of reviews. Health Quality Life Outcomes. 2017; 15(1): 220. doi: 10.1186/s12955-017-0778-6 29141645 PMC5688651

[34] Barger D, Hessamfar M, Neau D, Vareil MO, Lazaro E, Duffau P, et al. Assessing the psychometric properties of the French WHOQOL-HIV BREF within the ANRS CO3 Aquitaine Cohort’s QuAliV ancillary study. Health Quality Life Outcomes. 2020; 18(1), 220. doi: 10.1186/s12955-020-01451-8 32650781 PMC7350695

[35] Pereira M, Martins A, Alves S, Canavarro MC. Assessing quality of life in middle-aged and older adults with HIV: psychometric testing of the WHOQOL-HIV-Bref. Quality of Life Research. 2014; 23(9): 2473–2479. doi: 10.1007/s11136-014-0707-7 24791929

[36] Eurostat. Glossary: EU 2020 Strategy. 2018a [Cited 2022 December 21]. Available from: https://ec.europa.eu/eurostat/statistics-explained/index.php?title=Glossary:EU_2020_Strategy

[37] Spanish National Statistics Institute. (2020). Indicadores de Calidad de Vida. Renta media y mediana. 2020 [Cited 2022 December 15]. Available from: https://www.ine.es/ss/Satellite?blobcol=urldata&blobheader=application%2Fpdf&blobheadername1=Content-Disposition&blobheadervalue1=attachment%3B+filename%3D1_1_1__Renta_media_y.pdf&blobkey=urldata&blobtable=MungoBlobs&blobwhere=751%2F767%2F111_2021.pdf&ssbinary=true

[38] Eurostat. (2018b). Glossary: Material deprivation. 2018b [Cited 2022 December 21]. Available from: https://ec.europa.eu/eurostat/statistics-explained/index.php?title=Glossary:Material_deprivation

[39] Fuster-Ruiz de Apodaca MJ, Molero F, de Montes LG, Agirrezabal A, Vitoria A. HIV- and AIDS-related stigma: psychosocial aspects in a representative Spanish sample. Spanish Journal of Psychology. 2013; 16: E30. doi: 10.1017/sjp.2013.52 23866225

[40] Brennan DJ, Emlet CA, Brennenstuhl S, Rueda S. Socio-demographic profile of older adults with HIV/AIDS: gender and sexual orientation differences. Canadian Journal on Aging. 2013; 32(1): 31–43. doi: 10.1017/S0714980813000068 23521923

[41] Caliari JS, Reinato LAF, Pio DPM, Lopes LP, Reis RK, Gir E. Quality of life of elderly people living with HIV/AIDS in outpatient follow-up. Revista Brasileira de Enfermagem. 2018; 71(suppl 1): 513–522. doi: 10.1590/0034-7167-2017-0127 29562006

[42] Catalan J, Tuffrey V, Ridge D, Rosenfeld D, Hall Team. What influences quality of life in older people living with HIV? AIDS Research and Therapy. 2017; 14(1): 22. doi: 10.1186/s12981-017-0148-9 28400851 PMC5387225

[43] Brañas F, Sánchez-Conde M, Carli F, Menozzi M, Raimondi A, Milic J, et al. Sex Differences in People Aging With HIV. Journal of Acquired Immune Deficiency Syndromes. 2020; 83(3): 284–291. doi: 10.1097/QAI.0000000000002259 32032279

[44] Murphy DA, Roberts KJ, Herbeck DM. HIV-positive mothers with late adolescent/early adult children: "empty nest" concerns. Health Care Women International. 2012; 33(4): 387–402. doi: 10.1080/07399332.2012.655395 22420679 PMC3327642

[45] Yousuf A, Musa R, Isa MLM, Arifin SRM. Anxiety and Depression Among Women Living with HIV: Prevalence and Correlations. Clinical Practice & Epidemiology in Mental Health. 2020; 16, 59–66. doi: 10.2174/1745017902016010059 32742296 PMC7372730

[46] Waldron EM, Burnett-Zeigler I, Wee V, Ng YW, Koenig LJ, Pederson AB, et al. Mental Health in Women Living With HIV: The Unique and Unmet Needs. Journal of the International Association of Providers of AIDS Care. 2021; 20. doi: 10.1177/2325958220985665 33472517 PMC7829520

[47] Kalichman S, Shkembi B, Hernandez D, Katner H, Thorson KR. Income Inequality, HIV Stigma, and Preventing HIV Disease Progression in Rural Communities. Prevention Science. 2019; 20(7): 1066–1073. doi: 10.1007/s11121-019-01013-5 30955136 PMC7000177

[48] Walcott M, Kempf MC, Merlin JS, Turan JM. Structural community factors and sub-optimal engagement in HIV care among low-income women in the Deep South of the USA. Culture, Health & Sexuality. 2016; 18(6): 682–694. doi: 10.1080/13691058.2015.1110255 26670722 PMC6047529

[49] Fumaz CR., Larrañaga-Eguilegor M, Mayordomo-López S, Gómez-Martínez S, González-García M, Ornellas A, et al. Health-related quality of life of people living with HIV infection in Spain: a gender perspective. AIDS Care. 2019; 31(12): 1509–1517. doi: 10.1080/09540121.2019.1597959 30917676

[50] Ruiz-Algueró M, Hernando V, Marcos H, Gutiérrez G, Pérez-Elías MJ, López-Bernaldo de Quirós JC, et al. Self-rated health among people living with HIV in Spain in 2019: a cross-sectional study. BMC Infectious Diseases. 2021; 21(1): 129. doi: 10.1186/s12879-021-05815-3 33516173 PMC7847002

[51] Kostev K. Sex Differences in People Aging With HIV in Germany. Journal of Acquired Immune Deficiency Syndromes. 2020; 84(3): e11. doi: 10.1097/QAI.0000000000002349 32195748

[52] Terrence Higgins Trust. Uncharted Territory. A report into the first generation growing older with HIV. 2017 [Cited 2023 March 2]. Available from: https://www.tht.org.uk/sites/default/files/2018-03/uncharted_territory_final_low-res.pdf

[53] de la Fuente A. The economic consequences of Covid in Spain and how to deal with them. Applied Economic Analysis. 2021; 29(85): 90–104. 10.1108/AEA-11-2020-0158

[54] Albrecht G, Fitzpatrick R, Scrimshaw S. Handbook of Social Studies in Health and Medicine. 1st ed. SAGE Publications; 2000

[55] Kim S, Egerter S, Cubbin C, Takahashi ER, Braveman P. Potential implications of missing income data in population-based surveys: an example from a postpartum survey in California. Public Health Reports. 2007; 122(6): 753–763. doi: 10.1177/003335490712200607 18051668 PMC1997243

[56] The Lancet Healthy Longevity. Ageing with HIV. The Lancet Healthy Longevity. 2022: 3(3): e119. doi: 10.1016/S2666-7568(22)00041-1 36098283

[57] Nanna MG, Chen ST, Nelson AJ, Navar AM, Peterson ED. Representation of Older Adults in Cardiovascular Disease Trials Since the Inclusion Across the Lifespan Policy. JAMA Internal Medicine. 2020; 180(11): 1531–1533. doi: 10.1001/jamainternmed.2020.2750 32897289 PMC7489390

[58] Prendki V, Tau N, Avni T, Falcone M, Huttner A, Kaiser L, et al. A systematic review assessing the under-representation of elderly adults in COVID-19 trials. BMC Geriatrics. 2020; 20(1): 538. doi: 10.1186/s12877-020-01954-5 33342426 PMC7749979

