# Supplementary material for: Sex differences in health-related quality of life and poverty risk among older people living with HIV in Spain: A cross-sectional study
Source: PLoS One. 2024 May 7;19(5):e0301335. doi: 10.1371/journal.pone.0301335 (PMC11075874; doi:10.1371/journal.pone.0301335)
Supplement: S2 File — (PDF) [file pone.0301335.s002.pdf]

Psychometric properties of the WHOQoL-HIV BREF questionnaire

In Table S2, we compare the psychometric properties of the WHOQoL-HIV BREF questionnaire from the Spanish validation of the questionnaire [1] with those in our study. We found that Cronbach's alpha (α) coefficients were acceptable (α>0.70) in all domains with the exception of spirituality/personal beliefs (α=0.69). Overall, McDonald's omega (ω) coefficients were more favourable than α measurements. In our study, all domains reported ω values ≥0.81 – except the spirituality/personal beliefs one (ω=0.72). Overall, we found no noticeable differences between the psychometric properties found in the validation study and those in our study.

Table S2. Psychometric properties of the WHOQoL-HV BREF domains

	Cronbach's alpha (α)		McDonald's omega (ω)	
	Validation	Study sample	Validation	Study sample
Physical health	0.73	0.81	0.79	0.81
Psychological health	0.81	0.85	0.85	0.86
Level of independence	0.67	0.78	0.80	0.81
Social relationships	0.75	0.81	0.81	0.82
Environmental health	0.81	0.87	0.85	0.87
Spirituality/personal beliefs	0.61	0.69	0.62	0.72

References

1. Fuster-Ruiz de Apodaca MJ, Laguía A, Safreed-Harmon K, Lazarus JV, Cenoz S, Del Amo J. Assessing quality of life in people with HIV in Spain: psychometric testing of the Spanish version of WHOQOL-HIV-BREF. Health Quality Life Outcomes. 2019; 17(1): 144. https://doi.org/10.1186/s12955-019-1208-8
